# Subnational mapping of anaemia and aetiologic factors in the West and Central African region

**DOI:** 10.1017/S1368980024002222

**Published:** 2024-12-19

**Authors:** Kaleab Baye, Bayuh Asmamaw Hailu, Simeon Nanama, John Ntambi, Arnaud Laillou

**Affiliations:** 1 Center for Food Science and Nutrition, Addis Ababa University, Addis Ababa 1176, Ethiopia; 2 Research Center for Inclusive Development in Africa, Addis Ababa, Ethiopia; 3 Monitoring and Evaluation, Wollo University, Dessie, Ethiopia; 4 Nutrition Section, UNICEF West and Central Africa Region, Dakar, Senegal

**Keywords:** Anemia, Etiology, Women, Hemoglobinopathy, Infection, Malaria

## Abstract

**Objectives:**

Despite bold commitments to reduce anaemia, little change in prevalence was observed over the past decade. We aimed to generate subnational maps of anaemia among women of reproductive age (WRA), malaria transmission and hemoglobinopathies to identify priority areas but also explore their geographical overlap.

**Design:**

Using the most recent Demographic and Health Surveys (DHS), we first mapped anaemia clusters across sub-Saharan Africa and identified the West and Central Africa (WCA) as a major cluster. Geographic clusters with high anaemia and related aetiologic factors were identified using spatial statistics. Multilevel regression models were run to identify factors associated with any, moderate and severe anaemia.

**Settings:**

West and Central African countries (*n* 17).

**Participants:**

WRA (*n* 112 024) residing in seventeen WCA countries.

**Results:**

There was a significant overlap in geographical clusters of anaemia, malaria and hemoglobinopathies, particularly in the coastal areas of the WCA region. Low birth interval (0·86 (0·77, 0·97)), number of childbirth (1·12 (1·02, 1·23)) and being in the 15–19 age range (1·47 (1·09, 1·98)) were associated with increased odds of any anaemia. Unimproved toilet facility and open defecation were associated with any anaemia, whereas the use of unclean cooking fuel was associated with moderate/severe anaemia (*P* < 0·05). Access to healthcare facility, living in malaria-prone areas and hemoglobinopathies (HbC and HbS) were all associated with any, moderate or severe anaemia.

**Conclusion:**

Interlinkages between infection, hemoglobinopathies and nutritional deficiencies complicate the aetiology of anaemia in the WCA region. Without renewed efforts to integrate activities and align various sectors in the prevention of anaemia, progress is likely to remain elusive.

Globally, 30 % of non-pregnant women of reproductive age (WRA) were estimated to be anaemic in 2019^(1)^. The consequences of anaemia include poor physical performance, impaired cognitive function and adverse perinatal outcomes. Women living in low- and middle-income countries are disproportionally affected by anaemia, further hindering health and economic progress^(1)^. Recognising this, in 2012, the World Health Assembly (WHA) approved a commitment to halve the anaemia prevalence among WRA by 2025, which was later extended to 2030 to align with the Sustainable Development Goals^(2)^. Despite these bold commitments, the anaemia prevalence has shown little change over the past couple of decades^(1,3)^, questioning our understanding of the aetiology of anaemia and the appropriateness of the interventions put in place to achieve the targets.

The West and Central African (WCA) region has one of the highest prevalence of anaemia in WRA in the world. According to recent estimates, about 55 % of WRA in the WCA region are anaemic, and the region has witnessed little progress over the years^(1,4)^. Understanding the geographic distribution of any, moderate and severe anaemia and their aetiologic factors at subnational level is critical to effectively address and prioritise interventions aimed at preventing and treating anaemia^(5)^. Many earlier studies often equated anaemia to Fe deficiency, which led to wide-held beliefs among programme managers and implementers that anaemia is best prevented and even treated with Fe supplementation. Recent studies have reminded the complexity of the aetiology of anaemia, particularly in regions like WCA, where nutritional deficiencies, infections, malaria and hemoglobinopathies can overlap^(6,7)^.

The present study aimed to map any, moderate and severe anaemia, risk of malaria transmission, and hemoglobinopathies to explore geographical overlaps to guide decision on where to intervene and which package to provide. We then evaluated the association between anaemia (any and moderate/severe) and non-nutritional factors to inform complementary interventions, that if implemented along with existing nutritional interventions, can maximise anaemia reduction.

## Methods

### Overview and data source

First, we mapped high (any, moderate and severe) anaemia prevalence clusters for sub-Saharan Africa to identify high prevalence clusters in the continent. We then pooled data on anaemia and related factors from seventeen countries in WCA region, by considering data from the most recent (post-2010) Demographic and Health Surveys (DHS), yielding a sample of 112 024 WRA. Given the very limited temporal change in anaemia prevalence observed over the last decade in the region, we considered that the difference in survey years between countries is unlikely to affect comparability between countries.

Geo-referenced data related to hemoglobinopathies and malaria risk were extracted from the Malaria Atlas Project database (MAPAtlas)^(8)^. Data extracted from MAPAtlas were (i) sickle hemoglobin HbS allele frequency, (ii) hemoglobin C allele frequency (HbC), (iii) glucose-6-phosphate dehydrogenase (G6PD) and (iv) temperature suitable for malaria transmission index. Data for HbC, HbS, G6PD and temperature were downloaded in a spatial raster file from the MAPAtlas interface, and the raster files were imported to QGIS. Using the longitude and latitude coordinates of the DHS survey, we extracted the values of the geo-coordinates corresponding to each location and integrated them into our dataset for further analyses.

### Outcomes

The prevalence and case-load distribution of anaemia was mapped for the WCA region. Anaemia was categorised into any, moderate and severe anaemia, after adjusting Hb values for altitude and smoking. The following WHO recommended cut-offs were used to define the severity of anaemia: mild anaemia (11·0–11·9 g/dl), moderate anaemia (8·0–10·9 g/dl) and severe anaemia (< 8·0 g/dl). ‘Any anemia’ in this study is defined as the occurrence of anaemia at any severity level, including mild, moderate or severe forms. The DHS programme measures Hb concentrations in a small volume of capillary blood with the HemoCue 201+ or the 301+ system^(9)^. Overlaps between any anaemia, risk of malaria transmission and hemoglobinopathy clusters were identified and mapped. Individual, household and community-level factors associated with any and moderate/severe anaemia were identified.

### Statistical analyses

#### Spatial statistics

First, we applied a Kulldoruff scan statistics on anaemia data from sub-Saharan Africa using the most recent (post-2010) DHS^(10)^. Geographic areas of (any, severe and moderate) anaemia case distribution that is significantly different from what would have been obtained if the distribution was random were identified by a circular window. Confirmatory spatial analyses were run using SATScan by applying purely spatial Poisson scan statistics, and only clusters with statistically significant values (*P* < 0·05) were retained. Second, by selecting the WCA anaemia cluster, we focused our analyses on data from seventeen countries, for which hotspot maps were generated. The prevalence and case-load (density) of anaemia were estimated for unmeasured areas using ordinary kringing via the software SAGA GIS^(11)^.

### Univariate and multilevel logistic regression

Univariate logistic regression with any anaemia as an outcome was run. Variables with *P* < 0·02 were included in the multilevel logistic regression. Multilevel logistic regression was run to identify factors associated with clustering of (any) anaemia among WRA. Separate logistic regression models were run for ‘any’ and ‘moderate and severe’ anaemia. Considering the hierarchical nature of the DHS data, we run a multilevel logistic regression at the individual and community levels^(12)^. First, a null model (M0) that is without independent variables was run to measure random variability using the intracluster correlation. The intracluster correlation was used to determine cluster-level variation^(13)^. The second model (M1) was adapted to all lower-level (individual-level) factors; the third model (M2) was used for all higher-level household and individual factors; and the fourth model (M3) accounted for both lower and higher-level factors. The model goodness-of-fit was checked by the Akaike information criterion.

Given recent evidence of potentially issues of misclassification related to the use of capillary blood and hemocue for Hb measurement, we have run additional multilevel regression model on the Hb concentration as a dependent variable (see online supplementary material, Supplemental Table S3). Statistical analyses were conducted using Stata v14.

## Results

**Fig. 1:**
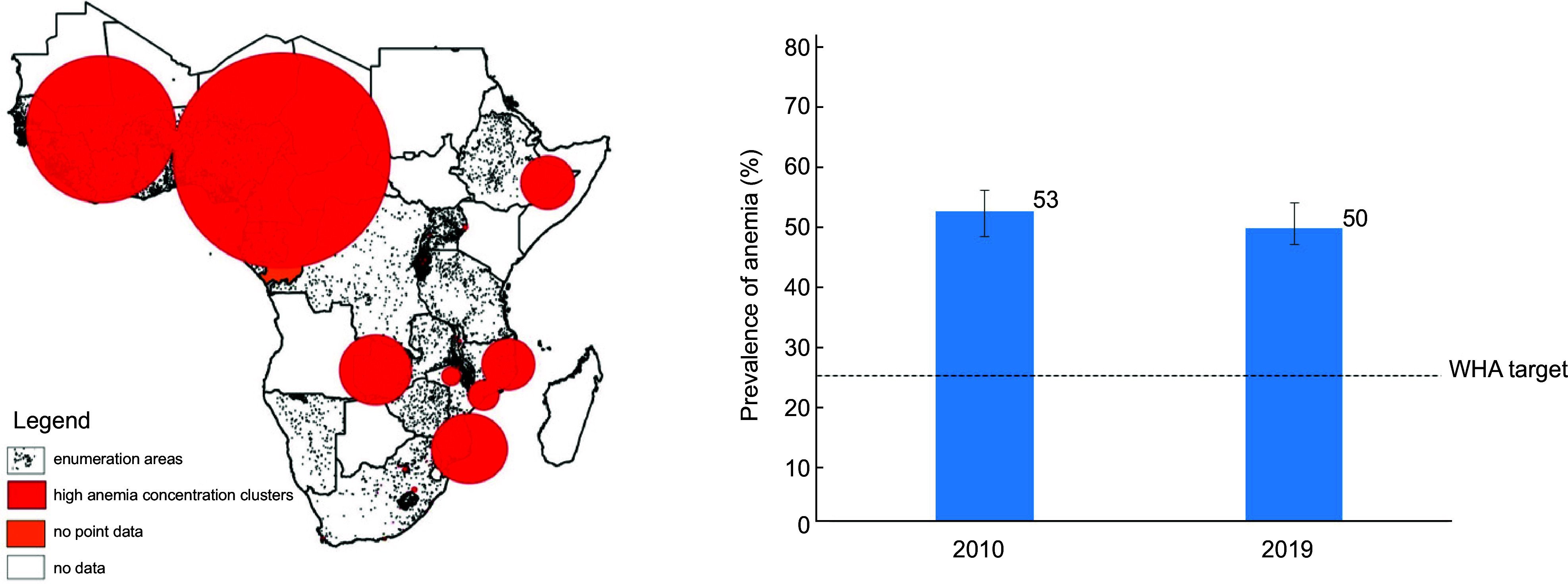
High anaemia clusters in sub-Saharan Africa (a) and the trend in anaemia prevalence (%) in West Africa for women of reproductive age. Values represented by the bar charts are median (quartile 1, quartile 3); data are from fifteen West African countries with post-2010 DHS data. WHA, World Health Assembly.

Our analyses were conducted on a total sample of 112 024 WRA, of which 56 346 (50·3%) were anaemic. Among the anaemic women, 39·4 % (20 854) had moderate anaemia and 2·4 % (1326) had severe forms of anaemia. Figure 1(a) shows maps with significant clusters of anaemia for the most recent DHS survey rounds. A total of eight significant clusters of any anaemia were identified for the sub-Saharan Africa region. The biggest anaemia concentration cluster was found in the WCA region and covered almost all the WCA countries. Figure 1(b) shows temporal trend in the anaemia prevalence between 2010 and 2019. A decrease of only three percentage points was observed for the WCA region. Further disaggregation of anaemia trend figures shows that these declines are largely driven by anaemia reductions observed in few countries like Ghana and Gambia (see online supplementary material, Supplemental Table S1). In contrast, countries like Mali, Niger and Sierra Leone have seen increases in the anaemia prevalence over the last decade.

**Fig. 2:**
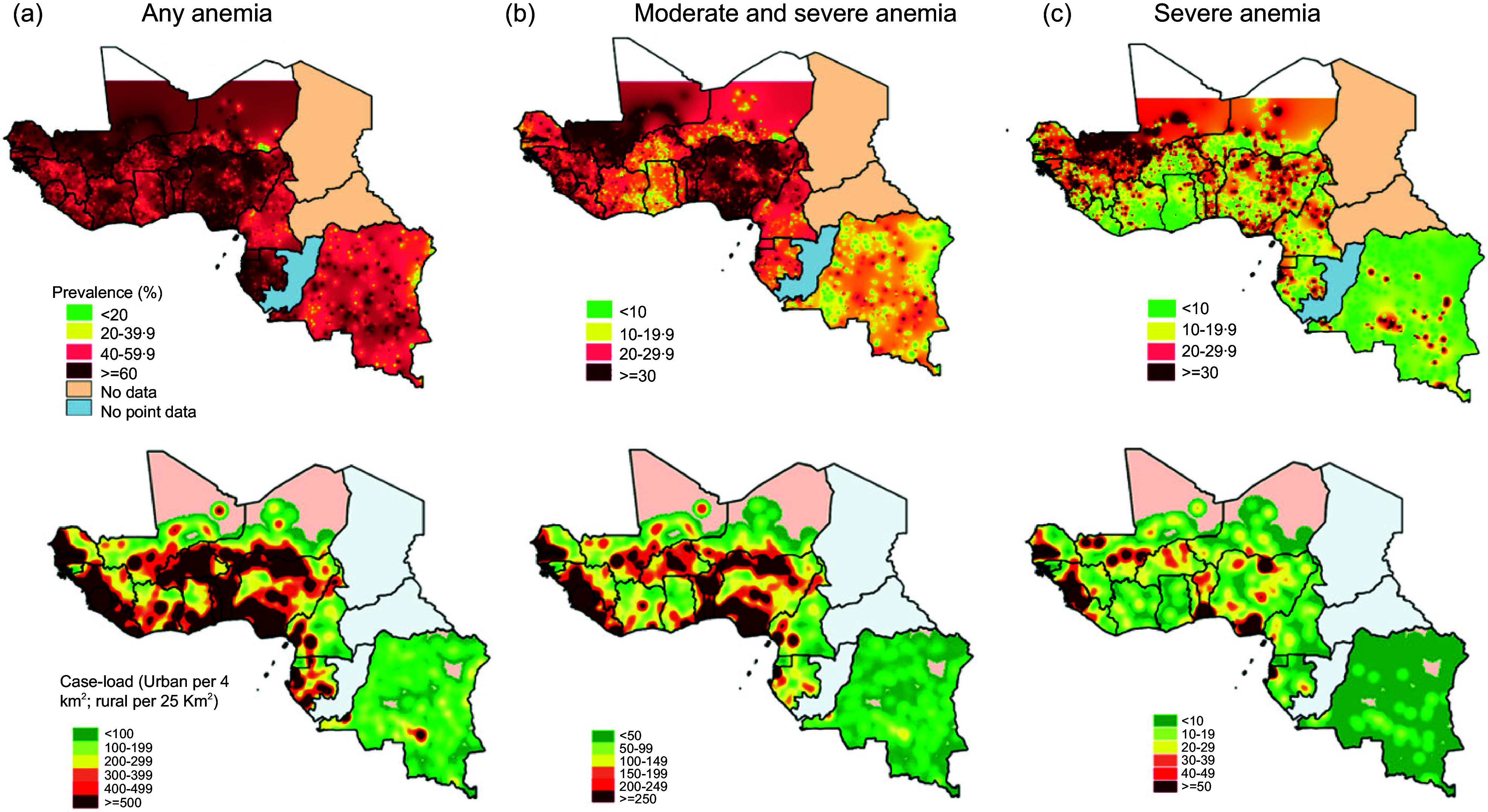
Mapping of anaemia (any) prevalence (a) and case-load density (b) in West and Central African countries, by severity.

Figure 2 presents the subnational distribution of the anaemia prevalence (A) and case-load/density (B), by severity. Most countries in the region had areas with prevalence of any anaemia exceeding 60 %, but countries like Nigeria, Gabon, Mali, Benin and Cote d’Ivoire had widespread areas with very high prevalence of any anaemia (> 60 %). However, when we considered moderate and severe forms of anaemia, regions with prevalence above > 30 % were concentrated in Nigeria, Benin, part of Sierra Leone, Liberia and Burkina Faso. Severe forms of anaemia were concentrated in Mali, Senegal, Guinea, Niger, Benin and pockets in Nigeria. The highest density of (any) anaemia cases were found in the coastal areas of the region, and most of Benin, Togo, Burkina Faso and northern Nigeria. Examples of subnational maps of countries in the region with varying prevalence of anaemia (see online supplementary material, Supplemental Fig. S1) illustrate the between and within-country heterogeneity for various forms (severity) of anaemia.

**Fig. 3:**
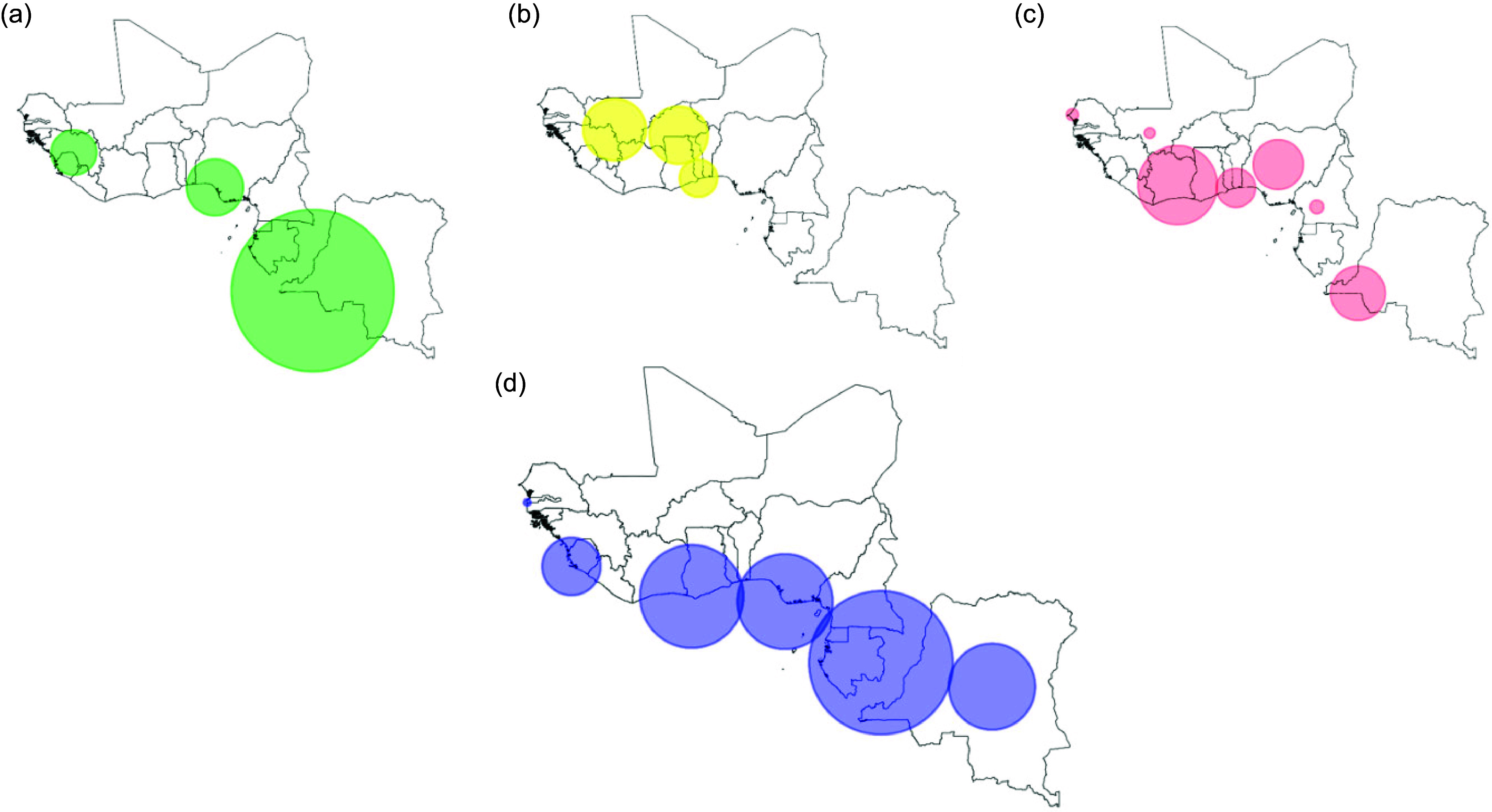
HbS (a), HbC (b), G6PD (c) and malaria (d) significant geographic clusters.

**Fig. 4:**
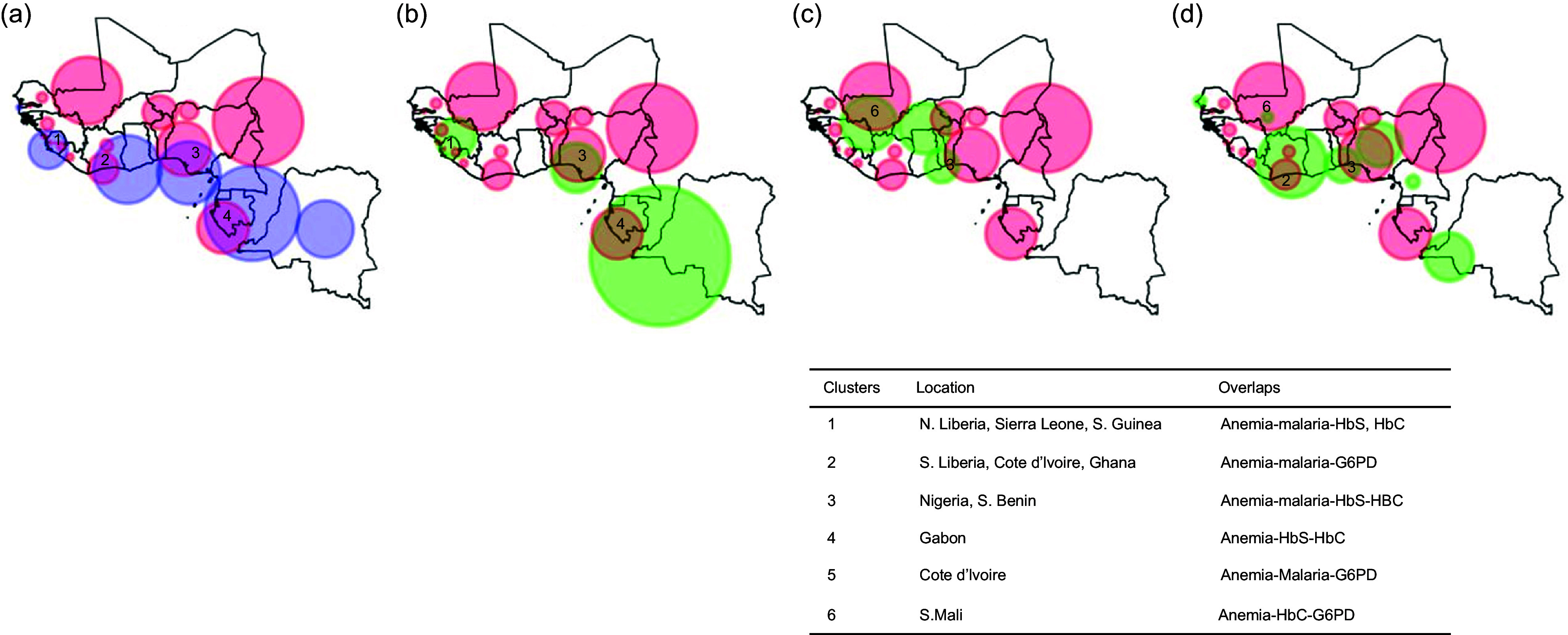
Overlap between clusters of temperature suitable for malaria (a), HbS (b), HbC (c), G6PD (d) and anaemia.

Figures 3 and 4 present maps showing concentration clusters of malaria and blood disorders such as HbS, HbC and G6PD. HbS concentration clusters were found in Nigeria, Benin, Guinea, Sierra Leone, Gabon and part of DRC. HbC was concentrated in Southern Mali, Guinea, Northern Cote D’Ivoire, Burkina Faso, Northern Ghana, Togo and Benin. The main G6PD concentration clusters were found in Cote D’Ivoire, Central Nigeria, Western DRC, Southern Benin and Togo. Many of the coastal areas of West Africa had temperatures suitable for malaria transmission. Six clusters where anaemia, malaria and blood disorders (HbS, HbC or G6PD) overlapped were identified, and these included the following countries: Liberia, Sierra Leonne, Guinea, Ghana, Côte d’ivoire, Nigeria and Benin. In Gabon and Mali, the anaemia clusters overlapped only with blood disorders.

**Fig. 5:**
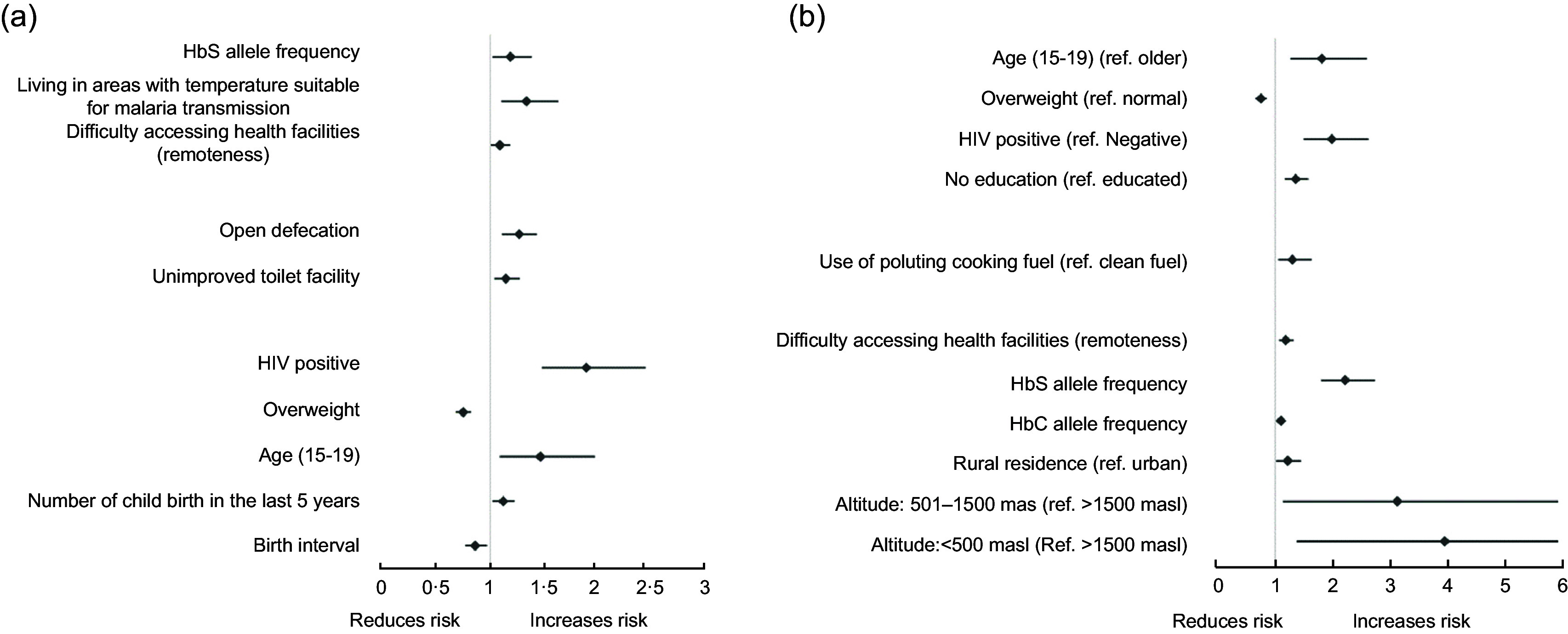
Factors significantly associated with any anaemia (a) and moderate/severe anaemia, adjusted multilevel regression model. All values are statistically significant (*P* < 0·05).

Figure 5 presents a forest plot showing factors significantly associated with any or moderate/severe forms of anaemia in the adjusted multilevel model. Detailed results of the model are presented in online supplementary material, Supplemental Table S2. A number of individual factors like low birth interval (< 24 months), number of childbirths over the last 5 years and being in the 15–19 age range (adolescent) was associated with increased odds of any anaemia. In contrast, overweight was associated with reduced odds of anaemia. Being adolescent and reported HIV-positive were factors that were significantly associated with increased odds of moderate/severe anaemia. In contrast, overweight was associated with reduced odds of moderate/severe anaemia. Among household/community factors, unimproved toilet facilities and open defecation were associated with any anaemia, whereas the use of polluting cooking fuel was associated with moderate/severe anaemia only.

Among environmental factors, difficulty of accessing the nearest healthcare facility, living in areas with temperature suitable for malaria and areas with high HbS allele frequency were associated with any anaemia. Among these, living in malaria-prone areas was not associated with moderate or severe forms of anaemia. HbC allele frequency, rural residence and living in altitude lower than 1500 masl were significantly associated with higher odds of moderate/severe forms of anaemia.

## Discussion

The present study aimed to characterise the geographic distribution of anaemia and identify social, environmental and health factors that contribute to the anaemia burden of WRA in West and Central Africa. Our study showed that the WCA region is home to the biggest anaemia cluster in sub-Saharan Africa. Subnational mapping of anaemia revealed that areas with high anaemia prevalence (> 50 %) were widespread. Higher risk for anaemia, malaria transmission and related blood disorders like sickle cell trait overlapped in the coastal areas of the WCA region. A number of individual household and environmental factors were associated with anaemia, suggesting that the health and WASH sectors should more prominently engage with the nutrition sector in the fight against anaemia.

Despite bold commitments to reduce the burden and prevalence of anaemia (e.g. Sustainable Development Goals), little progress has been observed over the last decade. Regions like the WCA constitute anaemia hotspot areas, with one of the world’s highest reported prevalence of anaemia. Our estimate from this study suggests that one out of every two women is anaemic, a finding in line with recent global estimates^(1)^. Only three percentage point reduction in anaemia prevalence was observed between 2010 and 2019, clearly indicating that efforts put towards anaemia prevention are either suboptimal, not effective or at times misguided by the believe that anaemia is solely Fe-related. Indeed, the main, if not the only, existing public health interventions directed towards anaemia prevention and treatment are Iron-Folic Acid (IFA) supplementation and malaria control, mirroring the two immediate factors that are nutritional deficiencies and diseases. Although these interventions are proven to be effective^(14)^, first they are not always effectively implemented at scale and in a convergent manner, particularly in the high anaemia clusters identified. Both interventions are implemented in silo, by distinct sectors (health and nutrition) and with no or limited interaction. Second, even when implemented well, these interventions would only address anaemia related to malaria or iron-folic acid deficiency; consequently, their impacts are attenuated in areas where the aetiology of anaemia is much more complex and involves a number of overlapping factors including underlying socio-economic contexts.

Our regression model identified a number of individual, household and environmental factors. Reproductive health related factors such as birth interval and parity, infections like HIV and malaria, and sanitation (i.e. open defecation and unimproved toilet facility) were all significant non-nutritional predictors of anaemia. Hemoglobinopathies (HbS and HbC) were widespread and were significantly associated with any anaemia for HbS, and moderate and severe forms for HbC. Although these are non-nutritional factors *per se*, they co-exist and interact with nutrition-related factors and can influence the effectiveness of existing nutritional interventions. For example, poor sanitation could increase the risk of helminth transmission, which can lead to hemolysis (e.g. hookworm) and poor absorption of nutrients^(6)^. Similarly, malaria and other infections/inflammation can reduce Fe absorption through the up-regulation of hepcidin^(15,16)^. In such context, the prioritisation of interventions may also change, with infection treatment being more favourable than Fe supplementation that could override the ‘natural’ defence mechanism that sequesters Fe to make it less accessible to the infectious agent^(17,18)^. Also equally important would be to prevent infections and inflammation through better WASH, living conditions and the prevention of indoor pollution related to the use of cooking fuel^(5)^.

In line with earlier studies, our study confirmed that blood disorders, malaria endemic areas and anaemia concentration clusters overlap^(19)^. Such overlap is expected as blood disorders like sickle cell are known to induce anaemia that was reported to provide protection against severe malarial anaemia, high density parasitemia and reduce all-cause mortality^(20)^. However, sickle cell trait/disease has also been associated with nutrient deficiencies, including those of Zn, vitamin D and folate. Depending on the severity of the sickle cell condition, nutritional interventions may have contrasting health impact. For example, prophylactic dosage of folic acid is helpful and currently used in the management of HbS^(21)^, whereas Fe supplementation can have adverse outcomes by reversing the protection conferred by sickle cell and anaemia against malaria and other infections^(22)^. Indeed, the British Society of Hematology guideline suggests that *‘Iron supplementation should be given to women with proven iron deficiency (serum ferritin < 30 μg/l); it should not be given empirically as for anaemic women without haemoglobinopathy’.* Therefore, considering the risk of multiple micronutrient deficiencies, the risks associated with Fe supply in face of the high malaria infections and sickle cell traits, perhaps a low Fe-containing multiple micronutrient supplementation may be a better and safer alternative to IFA in clusters where anaemia overlap with sickle cell trait and malaria. However, this warrants a study of its own.

The present study has a number of limitations that needs to be considered when interpreting the findings. First, due to the cross-sectional nature of the surveys, the relationships reported in this study should be interpreted not as a causal relationship, but as associations. Second, although a comprehensive list of non-nutritional factors was included in our study, given the complex and multifaceted cause of anaemia, these may not be complete. Third, Hb data from the DHS are from capillary blood whose accuracy has been questioned in recent years. Fourth, because of the intricate relationship between non-nutritional and nutritional causes of anaemia, teasing out their respective share was challenging, but rather our finding suggest that these two broad categories of aetiologic factors should be addressed together. Notwithstanding the above limitations, the present study provides a unique and comprehensive assessment of the relationship between anaemia, reproductive health, infection, hemoglobinopathy and other related factors.

The identification and mapping of clusters can help prioritise efforts and contribute to the design of more effective programmes where multiple factors complicating the management of the prevention of anaemia overlap. In view of the complexity of the aetiology of anaemia in the region, currently implemented prevention interventions are limited and simplistic. The aetiology of anaemia in this region is further complicated by the interlinkages between infection, hemoglobinopathies and nutritional deficiencies. Without renewed action to integrate efforts from various sectors, and recognising the additional complication caused by hemoglobinopathies in the WCA region, progress is likely to remain elusive. In this study, we have identified geographical areas (overlap clusters) where such integration can be prioritised. Studies on the most optimal treatment and mix of interventions are urgently needed, but the whole region is likely to benefit from heightened malaria and infection prevention and treatment, reproductive health interventions that help increase birth interval and improved nutrient intake through improved diets or nutritional supplementation. However, Fe-containing supplements may need to be contextually evaluated and adapted. Although a transition from IFA to a low Fe-containing micronutrient supplementation is likely to be beneficial in this context of widespread infection and sickle cell trait, this warrants to be studied urgently.

## Supporting information

Baye et al. supplementary material Baye et al. supplementary material

## Supplementary material

For supplementary material accompanying this paper visit https://doi.org/10.1017/S1368980024002222
