# Supplementary material for: Subnational mapping of anaemia and aetiologic factors in the West and Central African region
Source: Public Health Nutr. 2024 Dec 19;28(1):e6. doi: 10.1017/S1368980024002222 (PMC11736650; doi:10.1017/S1368980024002222)
Supplement: Baye et al. supplementary material [file S1368980024002222sup001.docx]

**Supplementary appendix**

**Subnational mapping of anemia and etiologic factors in the West and Central African Region**

Kaleab Baye (PhD), Bayuh Asmamaw Hailu (MSc), Simeon Nanama (PhD), John Ntambi (MPH), Arnaud Laillou (PhD)

**Contents**

[**Table S1** Description of the dataset used and estimates of anemia by severity 2](#_Toc135284805)

[**Table S2** Significant factors predicting anemia identified by multivariate logistic regression 3](#_Toc135284806)

[**Table S3** Multivariate linear regression predicting change in hemoglobin concentration 4](#_Toc135284807)

[**Figure S1** Country-level maps showing examples of between and within country variation in anemia 7](#_Toc135284808)

# **Table S1** Description of the dataset used and estimates of anemia by severity

|  |  | **Anemia prevalence (%)** | | | | | |  |  |
| --- | --- | --- | --- | --- | --- | --- | --- | --- | --- |
| **Country** | **Sample** | | **any** | **moderate/severe** | **moderate** | **severe** | **Year of survey** | | |
| Burkina Faso | 8,424 | | 48.83 | 15.07 | 13.98 | 1.09 | 2010 | | |
| Benin | 8,018 | | 57.73 | 32.11 | 30.24 | 1.87 | 2017/8 | | |
| Congo DR | 9,349 | | 38.37 | 8.76 | 8.42 | 0.34 | 2013/4 | | |
| Congo | 5,364 | | 54.16 | 11.58 | 11.32 | 0.26 | 2011/2 | | |
| Cote d'Ivoire | 4,589 | | 53.75 | 14.83 | 14.34 | 0.49 | 2011/2 | | |
| Cameroon | 6,829 | | 39.74 | 18.24 | 17.41 | 0.83 | 2018 | | |
| Gabon | 5,267 | | 60.58 | 19.27 | 18.42 | 0.85 | 2012 | | |
| Ghana | 4,644 | | 42.37 | 10.17 | 9.78 | 0.39 | 2014 | | |
| Gambia | 5,858 | | 44.28 | 18.48 | 17.28 | 1.2 | 2019/20 | | |
| Guinea | 5,217 | | 45.82 | 23.22 | 21.72 | 1.5 | 2018 | | |
| Liberia | 4,027 | | 44.51 | 21.41 | 20.63 | 0.78 | 2019/20 | | |
| Mali | 5,063 | | 63.41 | 38.54 | 34.22 | 4.32 | 2018 | | |
| Nigeria | 14,617 | | 57.81 | 30.01 | 28.42 | 1.59 | 2018 | | |
| Niger | 5,060 | | 45.8 | 13.09 | 12.23 | 0.86 | 2012 | | |
| Sierra Leone | 7,266 | | 46.47 | 23.5 | 21.95 | 1.55 | 2019 | | |
| Senegal | 7,906 | | 54.1 | 13.72 | 12.69 | 1.03 | 2017 | | |
| Togo | 4,525 | | 52.21 | 11.79 | 11.16 | 0.63 | 2017 | | |

# **Table S2** Significant factors predicting anemia identified by multivariate logistic regression

|  | **Any anemia (WRA only)** | **Moderate and severe anemia** |
| --- | --- | --- |
| **Individual factors** |  |  |
| Birth interval | 0.86(0.77,0.97)** |  |
| Number of child birth in the last 5 years | 1.12(1.02,1.23)** |  |
| Age (15-19) | 1.47(1.09,1.98)** | 1.82(1.27,2.62)** |
| Overweight | 0.75(0.68,0.82)** | 0.75(0.65,0.85)** |
| HIV positive | 1.90(1.48,2.45)** | 2.00(1.50,2.65)** |
| No education |  | 1.36(1.17,1.59)** |
| Unimproved toilet facility | 1.15(1.04,1.28)** |  |
| Use of polluting cooking fuel |  | 1.31(1.05,1.64)** |
| **Community and environmental factors** |  |  |
| Open defecation | 1.27(1.11,1.44)** |  |
| Difficulty accessing health facilities (remoteness) | 1.09(1.00,1.19)** | 1.19(1.07,1.33)** |
| Living in areas with temperature suitable for malaria transmission | 1.34(1.10,1.64)** |  |
| HbS allele frequency | 1.19(1.02,1.39)** | 2.24(1.81,2.77)** |
| HbC allele frequency |  | 1.10(1.05,1.16)** |
| Rural residence |  | 1.22(1.02,1.46)** |
| Altitude (501–1500 masl) |  | 3.16(1.14,8.78)** |
| Altitude (<500 masl) |  | 3.99(1.39,11.47)** |

Masl, meters above sea level

# **Table S3** Multivariate linear regression predicting change in hemoglobin concentration

|  | **Model 1**  **without cluster level factors** | | | **Model 2**  **with cluster level factors** | |  |
| --- | --- | --- | --- | --- | --- | --- |
|  | β | **95% CI** | **P-value** | β | **95% CI** | **P-value** |
| **(Intercept)** | 12.282 | [11.939,12.626] | 0.00 | 12.67 | [12.285,13.056] | <0.001 |
| **Individual level factors** | | | | | | |
| **Education** (ref. secondary and above |  |  |  |  |  |  |
| Primary | -0.06 | [-0.106,-0.014] | 0.01 | -0.06 | [-0.106,-0.014] | 0.01 |
| No education | -0.087 | [-0.136,-0.039] | 0.00 | -0.087 | [-0.136,-0.039] | <0.001 |
| **Age** (ref. 40–49) |  |  |  |  |  |  |
| 30–39 | 0.058 | [0.007,0.109] | 0.03 | 0.058 | [0.007,0.109] | 0.03 |
| 20–29 | 0.066 | [0.015,0.116] | 0.01 | 0.066 | [0.015,0.116] | 0.01 |
| 15–19 | -0.12 | [-0.191,-0.049] | 0.001 | -0.12 | [-0.191,-0.049] | 0.001 |
| **Maternity** (ref. neither pregnant nor breastfed) | |  |  |  |  |  |
| Pregnant | -1.23 | [-1.278,-1.182] | 0.00 | -1.23 | [-1.278,-1.182] | <0.001 |
| Breastfeed | 0.003 | [-0.031,0.038] | 0.85 | 0.003 | [-0.031,0.038] | 0.85 |
| **BMI** (ref. Not underweight |  |  |  |  |  |  |
| Underweight | -0.087 | [-0.139,-0.036] | 0.001 | -0.087 | [-0.139,-0.036] | 0.001 |
| **Place of delivery** (ref. Health facility ) |  |  |  |  |  |  |
| Home | -0.072 | [-0.115,-0.029] | 0.001 | -0.072 | [-0.115,-0.029] | 0.001 |
| **ANC** (ref. >=4) |  |  |  |  |  |  |
| <4 | -0.04 | [-0.075,-0.004] | 0.03 | -0.04 | [-0.075,-0.004] | 0.03 |
| **IFA supplementation during pregnancy** (ref. Yes) | |  |  |  |  |  |
| No | -0.001 | [-0.046,0.044] | 0.96 | -0.001 | [-0.046,0.044] | 0.96 |
| **Birth interval last 5 years** (ref. only 1) |  |  |  |  |  |  |
| >=24 | -0.023 | [-0.058,0.013] | 0.21 | -0.023 | [-0.058,0.013] | 0.21 |
| <24 | 0.012 | [-0.037,0.06] | 0.64 | 0.012 | [-0.037,0.06] | 0.64 |
| **Toilet facility** (ref. improved) |  |  |  |  |  |  |
| Unimproved | -0.026 | [-0.07,0.018] | 0.25 | -0.026 | [-0.07,0.018] | 0.25 |
| **Source of drinking water** (ref. improved) | |  |  |  |  |  |
| Unimproved | -0.02 | [-0.068,0.028] | 0.41 | -0.02 | [-0.068,0.028] | 0.41 |
| **Type of cooking fuel** (ref. clean fuel) |  |  |  |  |  |  |
| Polluting fuel | -0.011 | [-0.103,0.081] | 0.81 | -0.011 | [-0.103,0.081] | 0.81 |
| **Wealth quintile** (ref. richest) |  |  |  |  |  |  |
| Poorest | -0.161 | [-0.248,-0.074] | 0.00 | -0.161 | [-0.248,-0.074] | <0.001 |
| Second | -0.152 | [-0.234,-0.071] | 0.00 | -0.152 | [-0.234,-0.071] | <0.001 |
| Middle | -0.109 | [-0.184,-0.033] | 0.005 | -0.109 | [-0.184,-0.033] | 0.005 |
| Fourth | -0.049 | [-0.115,0.017] | 0.15 | -0.049 | [-0.115,0.017] | 0.15 |
| **Distance to health facility** (ref. not a big problem) | |  |  |  |  |  |
| Big problem | -0.03 | [-0.066,0.006] | 0.11 | -0.03 | [-0.066,0.006] | 0.11 |
| **Cluster level factors** | | | | | | |
| **Education** (ref. secondary and above |  |  |  |  |  |  |
| Primary |  |  |  | 0.004 | [-0.111,0.118] | 0.95 |
| No education |  |  |  | -0.317 | [-0.425,-0.21] | <0.001 |
| **Age** (ref. 40–49) |  |  |  |  |  |  |
| 30–39 |  |  |  | 0.035 | [-0.117,0.188] | 0.65 |
| 20–29 |  |  |  | -0.029 | [-0.178,0.12] | 0.70 |
| 15–19 |  |  |  | -0.071 | [-0.283,0.141] | 0.51 |
| **Maternity** (ref. neither pregnant nor breastfed ) | |  |  |  |  |  |
| Pregnant |  |  |  | -1.309 | [-1.449,-1.168] | <0.001 |
| Breastfeed |  |  |  | -0.055 | [-0.153,0.043] | 0.27 |
| **BMI** (ref. Not underweight) |  |  |  |  |  |  |
| Underweight |  |  |  | -0.336 | [-0.486,-0.185] | <0.001 |
| **Place of delivery** (ref. Health facility ) |  |  |  |  |  |  |
| Home |  |  |  | -0.087 | [-0.171,-0.003] | 0.04 |
| **ANC** (ref. >=4) |  |  |  |  |  |  |
| <4 |  |  |  | -0.005 | [-0.095,0.084] | 0.91 |
| **IFA supplementation during pregnancy** (ref. Yes) | |  |  |  |  |  |
| No |  |  |  | -0.1 | [-0.198,-0.003] | 0.04 |
| **Birth interval last 5 years** (ref. only 1) |  |  |  |  |  |  |
| >=24 |  |  |  | -0.075 | [-0.179,0.029] | 0.16 |
| <24 |  |  |  | 0.005 | [-0.136,0.147] | 0.94 |
| **HH characteristics** | | | | | | |
| **Toilet facility** (ref. improved) |  |  |  |  |  |  |
| Unimproved |  |  |  | -0.041 | [-0.112,0.029] | 0.25 |
| **Source of drinking water** (ref. improved) | |  |  |  |  |  |
| Unimproved |  |  |  | 0.085 | [0.022,0.147] | 0.01 |
| **Type of cooking fuel** (ref. clean fuel) |  |  |  |  |  |  |
| Polluting fuel |  |  |  | -0.009 | [-0.15,0.132] | 0.90 |
| **Wealth quintile** (ref. richest) |  |  |  |  |  |  |
| Poorest |  |  |  | -0.234 | [-0.378,-0.09] | 0.001 |
| Second |  |  |  | -0.196 | [-0.327,-0.065] | 0.003 |
| Middle |  |  |  | -0.182 | [-0.299,-0.066] | 0.002 |
| Fourth |  |  |  | -0.028 | [-0.141,0.085] | 0.62 |
| **Distance to health facility** (ref. not a big problem) | |  |  |  |  |  |
| Big problem |  |  |  | -0.004 | [-0.068,0.06] | 0.91 |
| **Residence** (ref. urban) |  |  |  |  |  |  |
| Rural | -0.198 | [-0.239,-0.156] | <0.001 | 0.015 | [-0.037,0.068] | 0.57 |
| **G6PD** (Glucose-6-Phosphate Dehydrogenase (G6PD) deficiency frequency) | 0.013 | [0.002,0.025] | 0.02 | 0.01 | [-0.001,0.021] | 0.08 |
| **HbS** (Sickle Hemoglobin (HbS) Allele Frequency) | 0.016 | [-0.003,0.035] | 0.091 | 0.01 | [-0.008,0.028] | 0.29 |
| **HbC** (Hemoglobin C (HbC) Allele Frequency) | -0.022 | [-0.045,0.001] | 0.064 | 0 | [-0.022,0.022] | 0.10 |
| **Malaria** (T. suitability index for P. falciparum) | -0.007 | [-0.01,-0.004] | <0.001 | -0.008 | [-0.011,-0.005] | <0.001 |

The null model had a β [95% confidence interval] of 11.695 [11.675, 11.715] and P-value<0.001.

**
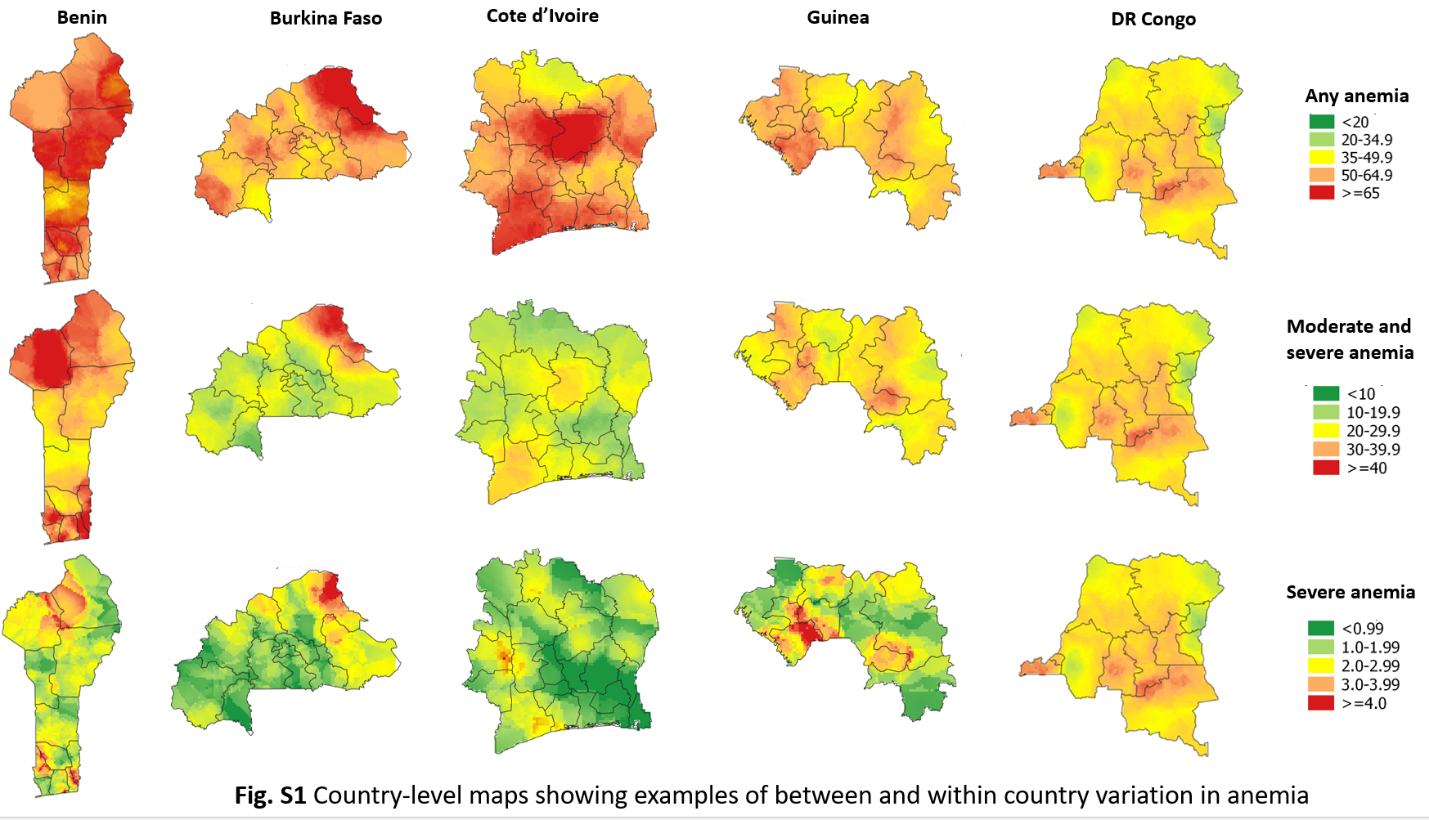
**

# **Figure S1** Country-level maps showing examples of between and within country variation in anemia
